# Size-Dependent Toxicity of Silver Nanoparticles to Bacteria, Yeast, Algae, Crustaceans and Mammalian Cells *In Vitro*

**DOI:** 10.1371/journal.pone.0102108

**Published:** 2014-07-21

**Authors:** Angela Ivask, Imbi Kurvet, Kaja Kasemets, Irina Blinova, Villem Aruoja, Sandra Suppi, Heiki Vija, Aleksandr Käkinen, Tiina Titma, Margit Heinlaan, Meeri Visnapuu, Dagmar Koller, Vambola Kisand, Anne Kahru

**Affiliations:** 1 Laboratory of Environmental Toxicology, National Institute of Chemical Physics and Biophysics, Tallinn, Estonia; 2 Institute of Physics, Faculty of Science and Technology, University of Tartu, Tartu, Estonia; 3 Biomineral Research Group, Medical Research Council Human Nutrition Research, Cambridge, United Kingdom; Texas A&M University, United States of America

## Abstract

The concept of nanotechnologies is based on size-dependent properties of particles in the 1–100 nm range. However, the relation between the particle size and biological effects is still unclear. The aim of the current paper was to generate and analyse a homogenous set of experimental toxicity data on Ag nanoparticles (Ag NPs) of similar coating (citrate) but of 5 different primary sizes (10, 20, 40, 60 and 80 nm) to different types of organisms/cells commonly used in toxicity assays: bacterial, yeast and algal cells, crustaceans and mammalian cells *in vitro*. When possible, the assays were conducted in ultrapure water to minimise the effect of medium components on silver speciation. The toxic effects of NPs to different organisms varied about two orders of magnitude, being the lowest (∼0.1 mg Ag/L) for crustaceans and algae and the highest (∼26 mg Ag/L) for mammalian cells. To quantify the role of Ag ions in the toxicity of Ag NPs, we normalized the EC_50_ values to Ag ions that dissolved from the NPs. The analysis showed that the toxicity of 20–80 nm Ag NPs could fully be explained by released Ag ions whereas 10 nm Ag NPs proved more toxic than predicted. Using *E. coli* Ag-biosensor, we demonstrated that 10 nm Ag NPs were more bioavailable to *E. coli* than silver salt (AgNO_3_). Thus, one may infer that 10 nm Ag NPs had more efficient cell-particle contact resulting in higher intracellular bioavailability of silver than in case of bigger NPs. Although the latter conclusion is initially based on one test organism, it may lead to an explanation for “size-dependent“ biological effects of silver NPs. This study, for the first time, investigated the size-dependent toxic effects of a well-characterized library of Ag NPs to several microbial species, protozoans, algae, crustaceans and mammalian cells *in vitro*.

## Introduction

The novel properties of man-made nanoparticles (NPs), i.e., particles having at least one dimension between 1–100 nm, are attributed to their small size, increased specific surface area and concomitant surface display of their constituent atoms. These properties in turn translate into higher surface reactivity and display of new electronic, optical, quantum mechanical and magnetic properties of nanosize particles. The physico-chemical properties that are responsible for technological breakthroughs could also lead to increased bioavailability and toxicity of engineered NPs compared to corresponding microsize compounds [1]. Although some types of the nanomaterials are already produced in industrial amounts, the data on their toxicological implications are just emerging [2].

As the main concept of nanotechnologies lies in specific “nano“ effects of small sized particulates, it is reasonable to assume that size-dependent biological effects, e.g. toxicity, within the nano-scale range (i.e. between 1–100 nm) could also be proven by appropriate experiments. According to Thomson Reuters ISI WoS, since 1990 the number of papers on toxic effects of NPs has rapidly increased and currently about 3500 papers are published on that theme annually. The papers focusing specifically on size-dependent toxic effects started to emerge about 15 years later and are still relatively rare (Figure S1). In total, the search (see SI) term “size-dependent toxic* AND nano*” yielded altogether about 200 papers. Currently, most of the studies that report higher toxicity of NPs compared to their microsize counterparts have been conducted by comparing nano-and microsize particles, mostly metal oxides and/or silver. As an example, the remarkable difference between toxicity of nano- and microsize CuO has been demonstrated in case of bacteria, algae, crustaceans, ciliates, fish, yeasts and nematodes: CuO NPs were shown to be 16-fold more toxic to algae and 48-fold more toxic to crustaceans than microsize CuO [3]. On the other hand, ZnO NPs and microsize ZnO were of comparable toxicity for the above described organism groups [3] which may be due to significant release of Zn ions from ZnO particles (for review, see [4]).

In addition to the comparisons between micro- and nanosize compounds, there are already some (mostly recent) studies where the toxicity of small “libraries” of NPs (mostly metal oxides or silver) of different sizes has been evaluated (Table 1, Figure S2). It is interesting to note, that although the majority of the results prove the hypothesis of toxicity increase with decreased particle size there are also experimental data showing that smaller NPs either were less toxic or, of no size-dependent toxicity (Table 1). One reason for these contradictory data could be that the NPs used for the toxicity tests are not usually monodisperse so theoretically, causing toxic effects of different magnitude. Due to the scarcity of experimental data on size-specific toxic effects (Figure S1) and contradictory experimental results obtained so far (Table 1), it is not surprising that there is no clear understanding of the effects of particle size on toxicity and no consensus exists on nano-threshold in (eco)toxicity [5]. Consequently, nanoparticle (NP) size has not been included as an important parameter in toxicity prediction models (QSAR or QNAR models) [6] and if NP size is bigger than 15–20 nm, it is not considered to be an important variable in determining their biological effects [7] [8]. This suggests that 15 nm could be considered as the maximum size for the “NP” of biological impact. Indeed, the nanotoxicity threshold at about 10 nm was also experimentally demonstrated by De Jong et al. [9], who showed that among gold NPs with primary particle sizes of 10, 50, 100 and 250 nm, only 10 nm particles were distributed in almost all organ systems, such as blood, liver, spleen, kidney, testis, thymus, heart, lung and brain after intravenous administration. Yet, given the scarcity of data, it is vital that more high quality experimental data will be available to improve the understanding on size-related biological effects of NPs.

**Table 1:** Summary of currently available nanoparticle size-related toxicity data for selected (eco)toxicological test organisms.

Nanoparticles (size)	Toxicity endpoint and the test organisms	Results	Additional (mechanistic) information provided	Reference
***PAPERS SHOWING HIGHER TOXICITY OF SMALLER PARTICLES***				
	**BACTERIA**			
Gallic-acid stabilised Ag NPs (7 nm and 29 nm)	Antibacterial properties, *Escherichia coli* and *Staphylococcus aureus*	MIC of 7 nm NPs was 6.25 mg/L (*E. coli*) and 7.5 mg/L (*S. aureus*). 29 nm particles were less potent, MIC = 13 mg/L (*E. coli*) and 17 mg/L (*S. aureus*).	n.a.	[42]
PVP-Ag NPs (mean sizes 5, 15 and 55 nm)	Antibacterial properties, five anaerobic oral pathogenic bacteria and aerobic bacteria *Escherichia coli*	For anaerobic bacteria, 5 nm particles were most effective (MIC 25 mg/L). Also for *E. coli*: 5 nm the most effective (MIC 6 mg/L)	The higher toxicity in aerobic conditions compared to anaerobiosis may be due to higher release of Ag^+^ from Ag NPs.	[43]
Ag NPs (20, 50, 110 nm)	Viability, bacteria *Escherichia coli*	Viability of *E. coli* depended on particle size (dose-dependent response)	Dissolution of Ag ions from the surface of the particles causing toxicity.	[44]
	**BACTERIA AND CRUSTACEANS**			
Ag NPs (Branched PEI-coated 10±4.6 nm; citrate-coated 56±14 nm; PVP-coated 72±24 nm)	3 h inhibition of β-galactosidase, *Escherichia coli*; Acute toxicity (48 h immobilisation), *Daphnia magna*	Particle size, surface charge, and concentration dependent toxicity for both the test organisms was shown (AgNO_3_ > BPEI-Ag NP > Citrate-Ag NP > PVP-Ag NP). The 48 h LC_50_ values for *D. magna* were 0.41 µg/L (BPEI-Ag NPs), 2.88 (citrate Ag NPs) and 4.79 µg/L (PVP Ag NPs), the enzyme inhibition endpoint (*E. coli* assay) showed EC_50_ values of 305, 791 and 2040 µg/L, respectively.	Bacteria *E. coli*: negligible contribution of dissolved Ag ion to the observed toxicity of Ag NPs.	[45]
	**CRUSTACEANS**			
ZnO NPs (30 nm and 80–100 nm) and ZnO microsized particles (>200 nm)	Feeding inhibition, 48 h immobilisation and reproduction of *Daphnia magna*	The 48-h LC_50_ for immobilization ranged between 0.76 mg Zn L^−1^ for the ionic zinc and 1.32 mg Zn L^−1^ for ZnO NPs of 80 nm to 100 nm.	Toxicity was explained by solubilised ions (endpoints: immobilisation, reproduction)	[46]
	**MAMMALIAN CELLS**			
Ag NPs (10, 40 and 75 nm citrate coated, 10, 50 PVP coated)	Cytotoxicity (lactate dehydrogenase release and Alamar blue staining)	Only 10 nm Ag NPs, regardless of coating, were toxic to human lung cells BEAS-2B.	Toxicity of 10 nm particles was due to particle uptake and subsequent intracellular release of Ag^+^ ions	[28]
Ag NPs (20 and 110 nm PVP coated, 20 and 1000 nm citrate coated)	Cytotoxicity (MTS assay) and cellular uptake	Smaller NPs were more toxic and were taken up by BEAS-2B cells more efficiently than larger particles	Higher toxicity of smaller particles was due to higher specific surface area and consquently, dissolution	[47]
***PAPERS SHOWING LOWER TOXICITY OF SMALLER PARTICLES***				
	**BACTERIA**			
Dextrose-encapsulated Au NPs (25, 60, and 120 nm±5 nm)	Antibacterial properties (effect on the growth, morphology and ultrastructural properties) to Gram-negative (*E. coli*) and Gram-positive bacteria (*Staphylococcus epidermidis*)	Both 120-nm and 60-nm gold NPs inhibited the proliferation of *E. coli* in a concentration-dependent manner with MIC of 16 ⋅ 10^10^ and 16 ⋅ 10^11^ NPs/mL, respectively. The 25-nm NPs did not significantly affect the proliferation of *E. coli* even at concentration as high as 128 ⋅ 10^12^ NPs/mL.	Bactericidal activity was mediated *via* disruption of the bacterial cell membrane which led to the leakage of cytoplasmic content.	[48]
***PAPERS SHOWING NO EVIDENT SIZE-DEPENDENT TOXICITY***				
	**BACTERIA**			
SiO_2_ NPs (15, 50 and 500 nm)	Antibacterial properties, *Bacillus subtilis*, *B. megaterium*, *E. coli* K12, *Listeria innocua* and *Pseudomonas fluorescens*.	No correlation between the size of SiO_2_ particles and effect on bacterial viability.		[49]
	**CYANOBACTERIA AND ALGAE**			
CeO_2_ NPs (<25 nm (N25), <50 nm (N50), N10, N60); microsize CeO_2_ (<5000 nm) (N50)	Inhibition of the self-luminescence, cyanobacterial recombinant strain *Anabaena* CPB4337. Inhibition of the growth, green alga *Pseudokirchneriella subcapitata*	*Anabaena*: 24 h EC_50_ from 0.27 to 6.3 mg/L; *P. subcapitata*: 72 h EC_50_ 2.4–29.6 mg/L. Algae: N50 was most toxic (EC_50_<1 mg/L; growth measured by OD). Order of the toxicity was N50 > N25 > N60 > N10 > B5000. Also, for Anabaena N50 was the most toxic NP (EC_50_<1 mg/L). Both organisms: no obvious relationship between primary size and toxic effect.	Uptake of NPs was not observed. Direct contact between NPs and cells was a prerequisite for toxic effect.	[50]
	**FISH**			
Ag NPs (20, 50, 110 nm)	Mortality, abnormal motility, zebrafish *Danio rerio*	NP-size-dependent response did not manifest in zebrafish when observing mortality for all Ag NP treatments	20 nm Ag NPs elicited the highest incidence of abnormal motility and induced slower development.	[44]

n.a. – not available.

The aim of the current paper was to generate and analyze a homogenous set of experimental toxicity data on silver NPs (Ag NPs) with similar coating (citrate) but of five different sizes ranging from 10 to 80 nm. Silver NPs were chosen for several reasons: (i) they are currently the NPs of the widest application area (e.g., as antimicrobials in medical equipment coatings, cosmetic products, textiles, sprays [10]; with the estimated global annual production of 22 tons [11]; **(ii)** among three biocidal NPs (CuO, ZnO and Ag), so far Ag NPs were shown to be the most toxic especially to crustaceans and algae (Table S1) – important representatives of the aquatic food webs [4]; **(iii)** there is a lack of comparable good quality toxicity data on Ag NPs (see the variability in currently available toxicity data in Table 1 and in Figure S3). In this paper, we present toxicity values of 10, 20, 40, 60 and 80 nm Ag NPs for bacteria *Escherichia coli* and *Pseudomonas fluorescens*, yeast *Saccharomyces cerevisiae*, algae *Pseudokirchneriella subcapitata*, crustacean *Daphnia magna* and murine fibroblast cell line Balb/3T3. We suggest that experiments conducted in the same laboratory using a well characterized library of monodisperse Ag NPs provide meaningful information on mechanisms of toxic action relating to different primary size of NPs and may be used as an input for quantitative toxicity modelling.

## Materials and Methods

### Chemicals

All the purchased chemicals were at least of analytical grade. Media components: yeast extract, tryptone, agar were from Lab M (Lancashire, UK) and peptone was from BD. Phosphate buffered saline (PBS), Dulbecco's Modified Eagle's Medium (DMEM) with high glucose content (4.5 g/L), Newborn calf serum (NBCS) and the mixture of penicillin and streptomycin (10 000 U/mL or 10 000 µg/mL, respectively) were purchased from Life Technologies. Neutral red (NR) was from Applichem. AgNO_3_ was purchased as 0.1 M solution from Fluka, 2,7- dichlorodihydrofluorescein diacetate from Invitrogen. Ultrapure water (UP water, pH 5.6±0.1) from MilliQ equipment (18 MΩ) was used throughout the study.

### Characteristics of the studied Ag nanoparticles

The characterisation and testing procedure is schematically depicted in Figure S4. Monodisperse Ag NP suspensions were purchased from MK Nano (Missisauga, Canada). According to the manufacturer, the NPs were stabilized with 2 mg/L of citrate and were supplied at 50–100 mg/L of Ag, in primary sizes of 10, 20, 40, 60 and 80 nm. Ag content in NP suspensions was determined by digestion in 1% HNO_3_ using Elan DRC Plus ICP-MS (Perkin Elmer). The morphology and the size of the particles were evaluated using transmission electron microscope (TEM) FEI-Philips Tecnai 10 operating at 80 kV and scanning electron microscope (SEM) FEI Helios NanoLab 600 (equipped with energy-dispersive X-ray spectroscopy (EDX) function) with accelerating voltage of 10 kV. Drops of aqueous Ag NP suspensions were placed onto Formvar/Carbon Coated copper-grids and silicon wafers for TEM and SEM/EDX analysis, respectively. The samples were allowed to dry at ambient conditions *prior* to imaging. EDX mapping was conducted using primary electron beam with acceleration voltage of 10 kV to detect Ag Lα X-ray fluorescence (2.98 keV). Particles on the obtained images were measured using ImageJ software [12]; average primary particle diameter was calculated from 20–30 particles.

Hydrodynamic diameter and surface charge (ζ-potential) of the Ag NPs (5–8 mg/L) in UP water and in test media used for bioassays were measured using Malvern Zetasizer (Nano-ZS, Malvern Instruments, UK). The pH of the purchased NP suspensions was determined by Thermo Orion 9863BN Micro pH Electrode (Thermo Scientific). UV-Vis absorption spectra of the Ag NPs were analysed on transparent 96-well polystyrene microplates (Greiner Bio-One) using plate spectrophotometer Multiskan (Thermo Scientific) at 300–800 nm wavelengths. Sedimentation of particles was determined by measuring 1 mL of NP suspensions in 1 cm polystyrene cuvettes over time (every 30 seconds from 0 till 60 minutes) using the Multiskan spectrophotometer at 420 nm.

To analyze the dissolution of Ag NPs in UP water, OECD 202 artificial freshwater (AFW) or algal growth medium (OECD 201), 1 mg Ag/L suspensions of Ag NPs or 0.01 mg Ag/L solution of AgNO_3_ (ionic control) in these media were incubated for 4, 24, 48 or 72 hours, respectively (the incubation time was selected according to the respective toxicity test protocol, see below). To analyze Ag NPs dissolution in mammalian cell culture medium, 10 mg Ag/L suspensions of Ag NPs or 1 mg Ag/L suspension of AgNO_3_ in cell culture medium were incubated for 24 hours. No test organisms/cells were used in dissolution experiments. Concentrations of Ag NPs or AgNO_3_ for the dissolution experiments were chosen according to the approximate EC_50_ values for different test organisms in the respective media. After incubation, dissolved Ag species were separated from particulate matter by ultracentrifugation at 390 000 g for 30 min (Beckman-Coulter ultracentrifuge L8-55 M) and dissolved Ag was determined from supernatants using GF-AAS in a certified laboratory of Tallinn University of Technology, Estonia, applying the standard EVS-EN ISO/IEC 17025∶2005. According to the calculations, under these conditions all Ag NPs and Ag-protein complexes with the molecular mass above 5 kDa should settle [13]. To prove that the majority of NPs had been removed by 30-min centrifugation at 390 000 g, the supernatants were also analysed using single particle (SP)-ICP-MS *via* an Elan DRC Plus (Perkin Elmer). By parallel analysis of the supernatant of the centrifuged NP suspensions and NP suspensions, very dilute original NP suspensions (5, 10, 50 and 100 pg/mL) and comparing the data with appropriate standards for Ag NPs (BBI, Cardiff, UK), it was possible to calculate the concentration of NPs in the centrifuged extracts. The data were evaluated, using the algorithm essentially by Peters et al. [14]. Clearly, the centrifugation had removed 99.4–99.9% of NPs and only 0.6, 0.15 and 0.02% of particles was present in supernatants of Ag 10, 20 and 80 nm NPs, respectively.

### Toxicity tests

#### 4 h acute toxicity assay using bacteria and yeast

The inhibitory effects of aqueous (prepared in UP water) suspensions of Ag NPs to bacteria *Escherichia coli* K12 (BW30270 from *E. coli* genetic stock Center, Yale University), *Pseudomonas fluorescens* OS8 [15] or *Saccharomyces cerevisiae* BY4741 from EUROSCARF collection (Institute of Microbiology, University of Frankfurt, Germany) was analysed. As a toxicity endpoint, the colony-forming ability of silver-exposed bacteria/yeast cells on agarized growth medium was used.

To start the microbial culture for the toxicity test, one colony was transferred from the agar plates to liquid growth medium and incubated overnight (30°C, 200 rpm). For growing the bacteria, NaCl-free LB medium (5 g/L yeast extract and 10 g/L tryptone; pH 7) and for yeast YPD medium (10 g/L of yeast extract, 20 g/L of peptone, 20 g/L of glucose; pH 6.6±0.1) was used. After overnight incubation, bacterial and yeast cultures were diluted 1∶50 into fresh medium and cultivated till the logarithmic growth phase. Then the cells were washed twice with UP water by centrifugation at 3500 g for 10 min and resuspended in UP water. The density of the microbial cell suspension in UP water was adjusted to 2–3⋅10^7^ cells/mL and used for the toxicity assays. For that, 100 µL of Ag NPs or AgNO_3_ dilution in UP water was first pipetted into microplate wells and then 100 µL of microbial suspension in UP water was added. The test plates with cells and chemicals were incubated at room temperature for 4 hours. Thereafter step-wise decimal dilutions from 10^1^ till 10^7^ from the exposed microbial cultures were prepared and 10–15 µL of such prepared solution was pipetted onto LB (bacterial assays) or YPD agar plate (yeast assay). To prepare agar plates, 1.5% agar was added to the bacterial and yeast growth media described above. The inoculated agar plates were incubated for 24–30 hours (bacterial assays) or for 48 hours (yeast assay) at 30°C and the grown colonies were counted. Colony forming units (CFU) per mL were calculated and EC_50_ values for Ag NPs and AgNO_3_ were calculated using GraphPad Prism program (GraphPad Software, Inc., USA) or Excel Macro Regtox [10]. To ensure that the exposure of bacterial/yeast cells in UP water for 4 hours had no influence on the viability of the cells we also determined the CFU values of non-exposed controls at the beginning and at the end of the 4-hour incubation period in UP water. No significant reduction of microbial cell numbers (CFU) was observed except for *E. coli* where the CFU/mL slightly decreased (from 3.2⋅10^7^ to 2.6⋅10^7^ i.e., by 18% upon 4-hour exposure).

#### 24 h cytotoxicity assay with mammalian fibroblasts

The inhibitory effect of Ag NPs and AgNO_3_ to murine fibroblasts Balb/3T3 (ATCC CCL-163) was analyzed using a Neutral Red (NR) assay. The cells were first grown in DMEM medium supplemented with 10% Newborn Calf Serum (NBCS), 100 U/mL penicillin and 100 µg/mL streptomycin mixture at 37°C and 5% CO_2_ till approximately 70% confluency. Then the cells were replated to transparent 96-well plates (5000 cells and 100 µL of cell culture medium per well) and further grown for 24 h at 37°C and 5% CO_2_. Next, the culture medium was removed and 100 µL of Ag NPs or AgNO_3_ diluted in fresh culture medium was added to the cells and the plates were incubated for 24 h at 37°C and 5% CO_2_. The plate was emptied again, the cells were washed with PBS and 100 µL of neutral red (NR) 5 mg/L solution in cell culture medium was added to all the wells. The plate was incubated for 3 h at 37°C and 5% CO_2_ and NR reagent was removed. The plate was washed with PBS and 100 µL of NR dilution reagent (50% ethanol, 1% glacial acetic acid) was added to the wells. This was followed by shaking the plate for 30 min at room temperature and measuring the absorbance at 540 nm using the plate reader Multiskan (Thermo Scientific). Abiotic values (NPs without fibroblasts) were always subtracted from the results obtained with Balb/3T3 cells. EC_50_ was calculated using GraphPad Software. Sodium dodecyl sulphate (SDS) was included every time in the assay as a positive control.

#### 72 h growth inhibition assay with algae *Pseudokirfchneriella subcapitata*

In general the OECD 201 algal growth inhibition test protocol was followed. The algal test medium (OECD 201; 15 mg NH_4_Cl, 12 mg MgCl_2_⋅6H_2_O, 18 mg CaCl_2_⋅2H_2_O, 15 mg MgSO_4_⋅7H_2_O, 1.6 mg KH_2_PO_4_, 0.08 mg FeCl_3_⋅6H_2_O, 0.1 mg Na_2_EDTA⋅2H_2_0, 0.185 mg H_3_BO_3_, 0.415 mg MnCl_2_⋅4H_2_O, 0.003 mg ZnCl_2_, 0.0015 mg CoCl_2_⋅6H_2_O, 0.00001 mg CuCl_2_⋅2H_2_O, 0.007 mg Na_2_MoO_4_⋅2H_2_O and 50 mg NaHCO_3_ per L; pH 8) was used. The *Pseudokirchneriella subcapitata* stock culture for the inoculation originated from the commercial test system Algal Toxkit F (MicroBioTests Inc., Nazareth, Belgium). 5 mL of algal test medium or Ag NP or AgNO_3_ dilutions in the algal test medium were inoculated with exponentially growing algal cells. The number of algae in the inoculum was counted under the microscope in the Neubauer haemocytometer and adjusted to yield 10 000 cells/mL in the sample after inoculation. For the assay, the algae were incubated in 20-mL glass vials on a transparent shaking table and constantly illuminated from below with Philips TL-D 38 W aquarelle fluorescent tubes at 24±1°C for 72 h. Only the bottoms of the vials received light with illuminance of 15 klx ±2 klx. During the 72 h the concentration of algal cells in the control culture increased at least 16 times and the pH did not change more than by 0.5 units. The algal biomass measurements were performed at least daily. The biomass was measured by chlorophyll fluorescence as described by Aruoja et al. [16]. Briefly, 50 µL of culture samples were transferred to 96-well plate, 200 µL of ethanol was added to each well and the plate was shaken for 3 h in the dark. Thereafter the fluorescence was measured with microplate fluorometer (excitation 440 nm, emission 670 nm; Fluoroskan Ascent, Thermo Labsystems, Finland). All the assays were run twice and each sample was analysed in duplicate with eight controls distributed evenly across the transparent table. EC_50_ values were calculated using Excel Macro Regtox [10].

#### 48 h acute toxicity assay with crustacean *Daphnia magna* (immobilization test)

The acute toxicity assay with crustacean *Daphnia magna* was performed according to OECD 202 guideline. Deviating from the standard test procedure, *D. magna* neonates used in the test were not originating from the laboratory culture but were hatched from the dormant ”eggs” (ephippia) purchased from MicroBioTests Inc. (Nazareth, Belgium) *prior* to testing. OECD 202 artificial freshwater (AFW) containing 294 mg CaCl_2_⋅2H_2_O, 123.25 mg MgSO_4_⋅7H_2_O, 64.75 mg NaHCO_3_, 5.75 mg KCl per L, pH = 7.8±0.2 was used as the control medium as well as the diluent for Ag NPs and AgNO_3_. Five *D. magna* neonates (less than 24 h old) were added to 10 ml of test sample and each concentration was analysed in four replicates, i.e. in total, 20 neonates per concentration were used. Test vessels with the exposed neonates were incubated in the dark at 20°C for 48 hours. The immobilization of the crustaceans was recorded after 24 and 48 h. The daphnids not able to swim within 15 seconds after gentle agitation of the test medium were considered immobilized (dead). EC_50_ values were calculated using GraphPad Prism program. Each NP/chemical was analyzed in at least two independent experiments.

#### Analysis of the bioavailable fraction of the studied silver preparations to recombinant *Escherichia coli* Ag-sensor

Bioavailability of Ag was analyzed by sensor bacteria *Escherichia coli* MC1061(pSLcueR/pDNPcopAlux), which is a Cu- and Ag-induced recombinant bioluminescent bacterium [17]. Bioluminescent response of this bacterium is mediated *via* Ag and Cu binding CueR protein which in the presence of intracellular silver or copper ions activates bioluminescence encoding genes and thus leads to an increase in light output. This increase in bioluminescence is concentration dependent and allows to calculate the amount of intracellular silver or copper ions [17]. *Prior* to analysis, sensor bacteria were cultivated overnight in NaCl-free LB medium (see above) with 10 µg/mL of tetracycline and 100 µg/mL of ampicillin. This was followed by 1∶50 dilution using new medium and sensor bacteria were further cultivated till logarithmic growth phase and then washed twice with UP water using centrifugation at 3500 g. The final number of sensor bacterial cells in the test was 10^7^/mL. For the bioavailability assay, 100 µL of Ag NPs or AgNO_3_ dilution in UP water was pipetted into the wells of a white 96-well microplate and then 100 µL of sensor bacteria suspension was added automatically by Luminometer Orion II (Berthold Detection Systems). Bioluminescence of the sensor bacteria was continuously registered during the first 10 sec of incubation and then once after 20 min and 4 hours of incubation. Bioluminescence induction of the bacteria was calculated as follows: 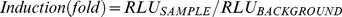
where RLU_SAMPLE_ is the bioluminescence of Ag-biosensor cells in the test sample (Ag-preparations in UP water) at the specified timepoint and RLU_BACKGROUND_ is the bacterial bioluminescence in UP water at the same time point. 2-fold induction was considered significant and was used in intracellular bioavailability calculations. Intracellular Ag was determined by using the log-log linear regression equations derived from the linear region of the dose-response curves of Ag-biosensor to AgNO_3_ and Ag NPs, whereas AgNO_3_ was considered 100% bioavailable and used as a standard. Each experiment was performed on two different days.

#### Analysis of the formation of abiotic reactive oxygen species (ROS) by the studied Ag formulations

To determine abiotic ROS, a procedure published by Priester et al. [18] was essentially followed. Briefly, 2,7-dichlorofluorescin diacetate (H_2_DCFDA) was dissolved in ethanol at 5 mM. 0.5 mL of 2,7-H_2_DCFDA was mixed with 10 mL of NaOH (final concentration 0.01 N) and incubated for 30 minutes in the dark. Then 40 mL of 25 mM sodium phosphate buffer (pH 7.2) was added and the mixture was further kept on ice. The final concentration of 2,7-H_2_DCFDA in the mixture was 0.05 mM. 80 µL of AgNO_3_ or Ag NPs suspension in UP water was pipetted into 96-well black microplate and 150 µL of the dye mixture was added. The fluorescence (excitation/emission  = 485/528±20 nm) was measured immediately and after 1 hour using Fluoroskan plate reader (Thermo Scientific). Increase in dye fluorescence was calculated using the following formula:
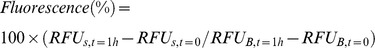
where RFU_S_ is fluorescence after exposure to the Ag sample and RFU_B_ is fluorescence in UP water (background fluorescence) at time point zero (t = 0) or after 1 hour (t = 1 h). Hydrogen peroxide was used as the positive control for ROS; in every assay, induction of 2,7-H_2_DCFDA fluorescence by 0.1–1.5% H_2_O_2_ was evident.

## Results and Discussion

### Characteristics of Ag nanoparticles

**Figure 1:**
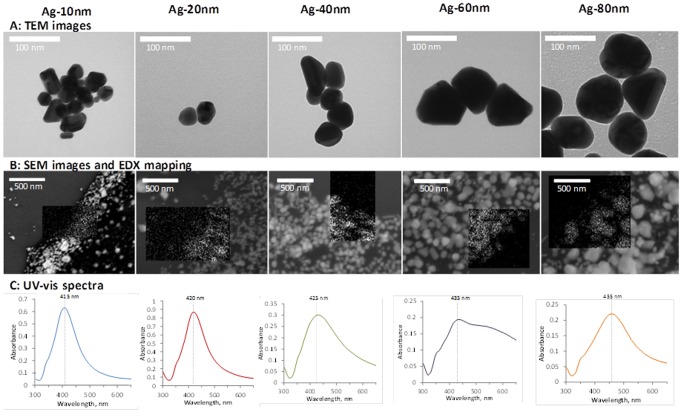
Electron microscopy images and UV-Vis absorption spectra of the studied citrate-stabilised Ag nanoparticles. A: TEM photos of the particles; B: SEM photos with EDX mapping; C: UV-Vis absorption spectra (Ag-10 nm 8 mg/L, Ag-20 nm 11 mg/L, Ag-40 nm, Ag-60 nm and Ag-80 nm 5 mg/L in ultrapure (UP) water. Maximum absorption is indicated with a vertical dotted line; wide absorption spectrum indicates polydispersity of the sample.

**Figure 2:**
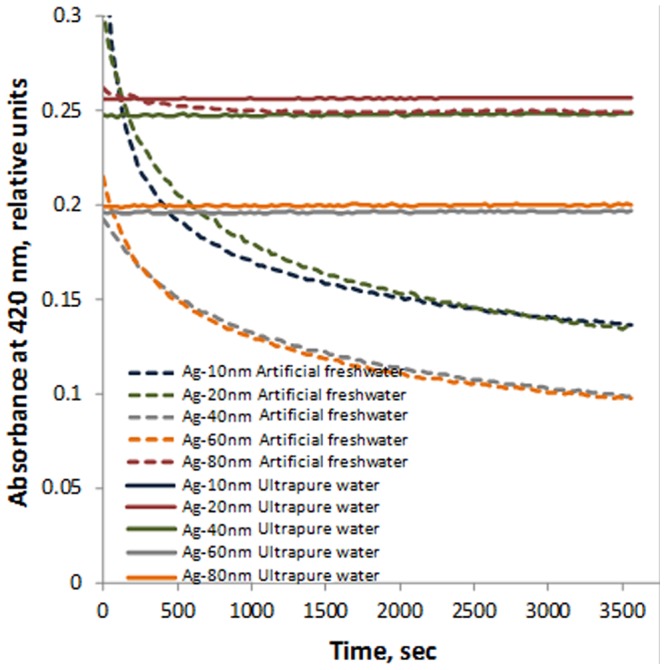
Sedimentation of Ag NPs in ultrapure water and in artificial freshwater during 60 Concentrations of Ag NPs were: Ag-10 nm 8 mg/L, Ag-20 nm 11 mg/L, Ag-40 nm, Ag-60 nm and Ag-80 nm 5 mg/L. Decreased absorption of light (420 nm) by Ag particles in artificial freshwater (test medium for *D. magna*) (dotted line) is due to settling over time. In ultrapure water (solid line) no decrease in absorption was observed.

Citrate-coated Ag NPs of different primary sizes (Figure 1, Table 2), purchased from MK Nano were used throughout the tests. The particles' stock suspensions provided by the producer were well dispersed. In UP water and in algal test medium the hydrodynamic size of the particles was close to their primary size and no sedimentation was observed (Figure 2, Table 2). In AFW (test medium for *D. magna*) the particle hydrodynamic diameter was enlarged so that the measurements were not possible (Table 2) and the particles settled in time (Figure 2). This was probably due to the presence of high levels of divalent cations that have been shown to increase Ag NP aggregation [19]. The particles surface charge (ζ-potential) in all the three test media was negative, varying from −15 mV till −29 mV (Table 2).

**Table 2:** Physico-chemical characteristics of the studied citrate-stabilised Ag nanoparticles.

Ag NPs	pH^a^	Primary size, nm		N^o^ of particles/mL^d^	Hydrodynamic size, nm (pdi)				ζ-potential, mV			
		b	c		UP water^e^	AFW^f^	Algal growth medium	Cell culture medium^g^	UP water^e^	AFW^f^	Algal growth medium	Cell culture medium g
Ag-10 nm	7.7	11.6±5.2	13	2.3⋅10^11^	6.00 (0.48)	n.a.*	9.70 (0.49)	68.6 (0.29)	−25	−20	−17	−9.59
Ag-20 nm	8.1	17.8±8	17	2.3⋅10^10^	11.0 (0.43)	n.a.*	14.00 (0.43)	76.0 (0.31)	−25	−15	−23	−10.0
Ag-40 nm	7.2	47.7±8	n.a.	n.a.	16.0 (0.28)	n.a.*	17.20 (0.28)	107 (0.26)	−24	−19	−27	−4.84
Ag-60 nm	7.2	56.5±9.6	n.a.	n.a.	58.0 (0.25)	n.a.*	110 (0.22)	162 (0.14)	−15	−18	−27	−8.29
Ag-80 nm	7.1	94.8±54	74	6⋅10^7^	68.0 (0.30)	n.a.*	89.0 (0.25)	153 (0.23)	−16	−15	−29	−9.20

a measured from Ag NPs stock suspension.

b measured from SEM images, 20–30 particles (see also Figure 1).

c according to single particle (SP)-ICP-MS.

d measured using SP-ICP-MS.

e UP water – ultrapure water.

f AFW – OECD 202 artificial freshwater used as test medium for crustaceans *Daphnia magna*.

g DMEM (cell culture medium) with 10% NBCS.

pdi – polydispersity index.

n.a. not available.

* the samples were too polydispersed.

### Toxicological profiles of Ag nanoparticles

**Figure 3:**
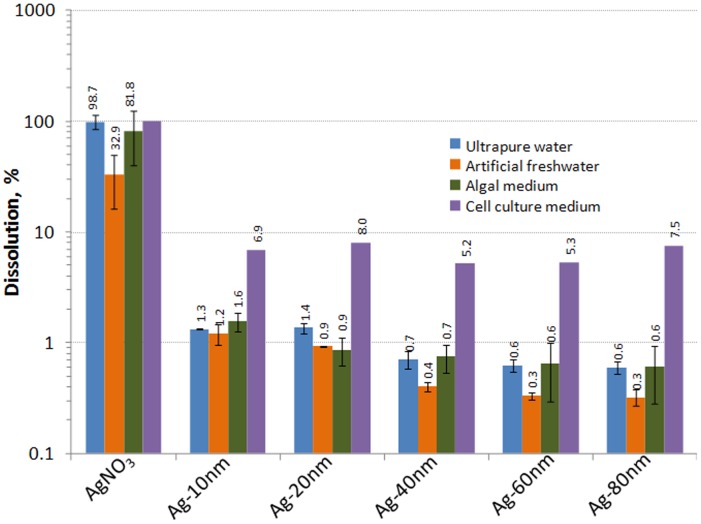
Dissolution (%) of Ag NPs in different test media. Ultrapure water was used as a solvent to mimic the dissolution in the bacterial and yeast assays, OECD 202 artificial freshwater was used for *Daphnia magna* assay, algal test medium for *Pseudokirchneriella subcapitata* and cell culture medium for Balb/3T3 murine fibroblast assay. Dissolved ionic Ag was measured after incubation of 1 mg/L Ag NPs or 0.01 mg/L AgNO_3_ for 4 hours (ultrapure water), 24 hours (cell culture medium), 48 hours (artificial freshwater) or 72 hours (algal medium), depending on the length of the toxicity assay. The results shown were measured from Ag NPs suspensions and AgNO_3_ solution after ultracentrifugation. These results were confirmed by single particle (SP)-ICP-MS according to which 1.4% of 10 nm Ag NPs, 1% of 20 nm Ag NPs and 0.5% of 10 nm Ag NPs had been dissolved in ultrapure water.

a **Dissolution of Ag NPs in different test media.** It has been suggested that the toxicity of particulate silver (as well as of ZnO and CuO) is largely due to the dissolved fraction [20] [21]. To study the contribution of the soluble fraction (Ag ions) to the toxicity of Ag NPs, we quantified the amount of Ag ions after incubation of Ag NPs in the test media used in the current study. The incubation time after which the particles were separated from dissolved ions by ultracentrifugation (Figure S4) was specific to the respective toxicity test. In general, the dissolution of ionic Ag from NPs was relatively low, usually less than 1% of the total Ag. In UP water, AFW and algal test medium the release of Ag ions depended clearly on the primary size of the Ag NPs (Figure 3, Table S2). For example, in UP water 1.3% of Ag was released as ions from 10 nm Ag NPs and only 0.6% of Ag was dissolved from 80 nm Ag NPs. Roughly similar results were obtained in algal test medium. Higher dissolution of smaller Ag NPs is coherent with the previous studies by [22] [23] and could be explained by their higher specific surface area.

In OECD 202 AFW that was used as test medium in crustacean immobilization assay the dissolution of Ag NPs was slightly lower compared to UP water and algal test medium yet followed the similar trend: smaller particles dissolved more (Figure 3). Lower dissolution of Ag NPs in AFW (Figure 3) could be explained by formation of insoluble Ag complexes as shown also by Miao et al. [24]. For example, in AFW, only 33% of the added AgNO_3_ was present as ionic form showing that most likely 80% of Ag ions settled during ultracentrifugation in the form of insoluble Ag.

Dissolution of Ag NPs in mammalian cell culture medium (DMEM with 10% serum) was remarkably different from what was observed in crustacean and algal test media. The fraction of dissolved Ag in cell culture medium was relatively high (5–7%; Figure 3) and no clear size-dependent dissolution was observed. The observed high dissolution of Ag NPs may be due to high organics content in this medium. As reported earlier for ZnO NPs [25] and CuO NPs [26] organic media components may promote metallic NPs' solubility.

b **Toxicity of Ag NPs to different test organisms/cells.** The EC_50_ values obtained in this work for Ag NPs as well as for Ag ions (Table 3) indicate that the most sensitive organism to silver NPs was crustacean *D. magna*, followed by algae *P. subcapitata*, bacteria *E. coli* and *P. fluorescens*, yeast *S. cerevisiae*, and finally, mammalian fibroblasts *in vitro* (Table 3, Figure S5). This order of sensitivity is in agreement with the literature data presented in Figure S3. The nominal Ag NPs-based EC_50_ data from the current study varied almost 3 orders of magnitude, ranging from 0.1 (*D. magna*) to 26 mg/L (fibroblasts) being again in agreement with previously reported literature data (Figure S3). These differences in EC_50_ values are likely due to differences in the used test media [27] as well inherent properties of the test organisms/cells (Table 3). Indeed, Blinova et al [27] showed that natural water (dissolved organic carbon content 5–35 mg C/L) mitigated the toxicity of the studied silver compounds to crustaceans *D. magna* and *T. platyurus* up to 8-fold compared to artificial freshwater. Different inherent sensitivity of organisms towards silver is evident by comparing the EC_50_ values of bacteria, yeast, algae and crustaceans. Although these assays were performed in mineral media or in UP water, toxicity of silver to these organisms was different (Table 3). Sensitivity pattern of the organisms towards silver agrees with that in Table S1, reported in Bondarenko et al [4].

**Table 3:** Nominal, dissolution- and bioavailability-corrected EC_50_ values (mg Ag/L) of different sized Ag NPs and ionic AgNO_3_ for various test organisms.

	Nominal EC_50_	EC_50_ corrected to Ag dissolution^a^	EC_50_ corrected to Ag bioavailability^b^
**Bacteria** (4 h; UP water; colony forming ability on agarised LB medium)			
*Escherichia coli*			
AgNO_3_	0.01±0.004	0.010±0.004	0.010±0.004
Ag-10 nm	0.27±0.2	0.004±0.0026	0.012±0.00011
Ag-20 nm	0.51±0.24	0.006±0.003	0.016±0.00009
Ag-40 nm	1.51±1.12	0.012±0.0091	0.016±0.0001
Ag-60 nm	2.56±1.6	0.017±0.011	0.020±0.00009
Ag-80 nm	2.96±1.83	0.019±0.012	0.020±0.00008
Average (for AgNPs):	1.56	0.011	0.016
*Pseudomonas fluorescens*			
AgNO_3_	0.02±0.007	0.02±0.007	0.02±0.007
Ag-10 nm	0.55±0.22	0.007±0.003	0.024±0.0009
Ag-20 nm	0.99±0.4	0.013±0.005	0.030±0.002
Ag-40 nm	2.12±1.11	0.015±0.008	0.022±0.005
Ag-60 nm	3.81±1.22	0.023±0.008	0.030±0.006
Ag-80 nm	5.25±1.82	0.031±0.01	0.035±0.009
Average (for AgNPs):	2.54	0.018	0.028
**Yeast** *Saccharomyces cerevisiae* (4 h; UP water; colony forming ability on agarised YPD medium)			
AgNO_3_	0.023±0.004	0.023±0.004	not calculated^c^
Ag-10 nm	1.53±0.51	0.016±0.0053	not calculated^c^
Ag-20 nm	2.72±0.8	0.029±0.0084	not calculated^c^
Ag-40 nm	7.28±2.42	0.049±0.163	not calculated^c^
Ag-60 nm	7.33±1.5	0.042±0.0085	not calculated^c^
Ag-80 nm	8.17±2.59	0.044±0.014	not calculated^c^
Average (for AgNPs):	5.4	0.036	not calculated^c^
**Algae** *Pseudokirchneriella subcapitata (72 h; algal growth medium; growth inhibition)*			
AgNO_3_	0.007±0.002	0.007±0.0023	not calculated^c^
Ag-10 nm	0.18±0.06	0.002±0.0009	not calculated^c^
Ag-20 nm	0.52±0.36	0.005±0.0037	not calculated^c^
Ag-40 nm	0.82±0.25	0.007±0.0022	not calculated^c^
Ag-60 nm	0.94±0.49	0.009±0.0044	not calculated^c^
Ag-80 nm	1.14±0.32	0.010±0.0027	not calculated^c^
Average (for AgNPs):	0.72	0.0067	not calculated^c^
**Crustacean** *Daphnia magna (48 h; artificial freshwater; immobilisation of neonates)*			
AgNO_3_	0.002±0.001	0.00039±0.00012	not calculated^c^
Ag-10 nm	0.010±0.014	0.00014±0.00006	not calculated^c^
Ag-20 nm	0.034±0.01	0.00031±0.00009	not calculated^c^
Ag-40 nm	0.141	0.00060	not calculated^c^
Ag-60 nm	0.168±0.0073	0.00053±0.00013	not calculated^c^
Ag-80 nm	0.218	0.00062	not calculated^c^
Average (for AgNPs):	0.11	0.0004	not calculated^c^
**Mammalian cells**: *murine fibroblast line BALB/3T3 (24 h; DMEM with 10% NBCS; viability)*			
AgNO_3_	1.7±0.57	1.70±0.58	not calculated^c^
Ag-10 nm	16.9±1.9	1.18±0.14	not calculated^c^
Ag-20 nm	22.0±1.3	1.76±0.11	not calculated^c^
Ag-40 nm	28.7±1.6	1.48±0.09	not calculated^c^
Ag-60 nm	30.9±2.1	1.65±0.12	not calculated^c^
Ag-80 nm	34.9±2.3	2.62±0.17	not calculated^c^
Average (for AgNPs):	26.7	1.73	not calculated^c^

a Dissolved ionic Ag was determined from the supernatant of ultracentrifuged nanoparticles suspension. See Table S2.

b Bioavailable Ag was analyzed using recombinant bioluminescent Ag-sensor bacteria *E. coli* MC1061(pSLcueR/pDNPcopAlux). See also Materials and Methods and Figure 6.

c not calculated as the bioavailability measurement was relevant only for bacterial cells.

**Figure 4:**
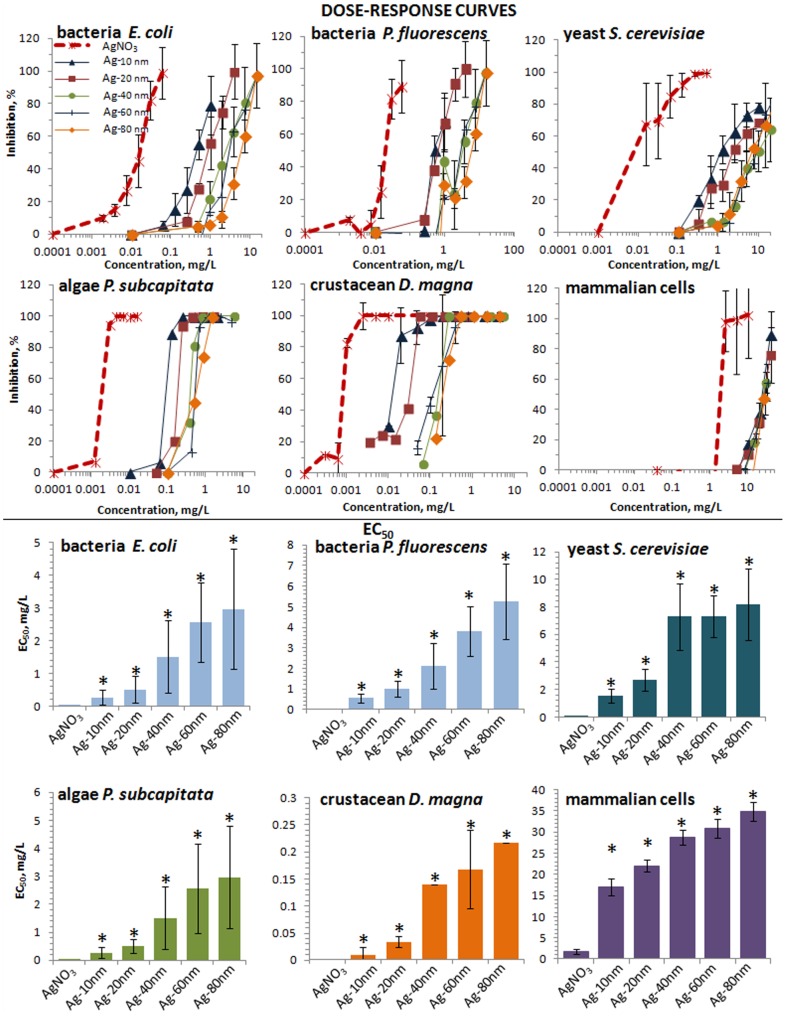
Dose-response curves and the respective EC_50_ values of Ag formulations to test organisms and cells. Upper panel (A–D): dose-response curves; lower panel (E–H): EC_50_ values. X-axis shows nominal Ag concentrations. * - significantly (p<0.05) different from EC_50_ value of AgNO_3_.

UP water – ultrapure water.

c **Particle-size dependent toxicity of Ag NPs towards different test organisms/cells.** When the toxic effect of Ag NPs towards each test organism/cell was analysed for each size separately, more subtle effects became evident. Nominal concentrations-based dose-response curves for Ag NPs with various primary sizes and AgNO_3_ for bacteria, yeast, crustaceans and algae are shown in Figure 4 upper panel and the respective EC_50_ values in the lower panel. For all the test organisms/cells, AgNO_3_ was the most toxic compound and, as a rule, the toxicity of Ag NPs decreased with increasing primary particle size. Similarities in the slopes of the dose-response curves for the test species for AgNO_3_ and Ag NPs varying in size (Figure 4, upper panel) also indicate that the tested Ag compounds may have a common mechanism of action, i.e. acting *via* solubilized silver.

**Figure 5:**
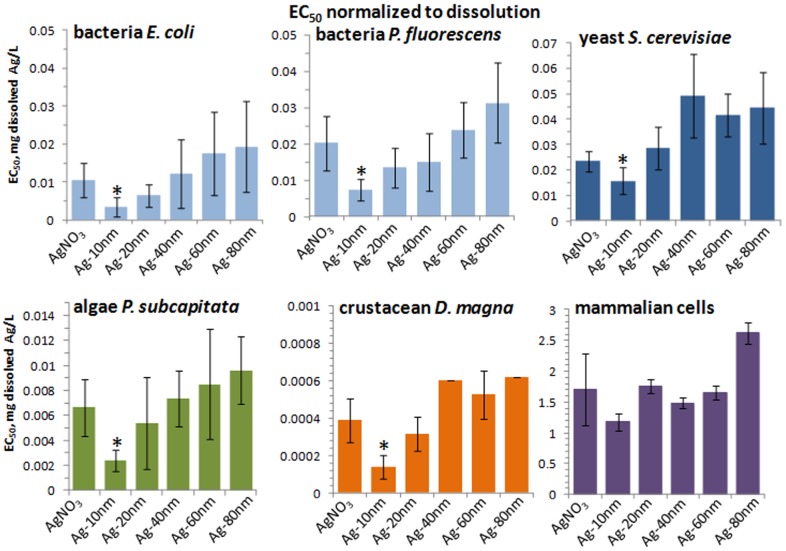
Dissolution-corrected EC_50_ values of 10–80 nm Ag NPs and AgNO_3_. EC_50_ values presented in Figure 4 (lower panel) were normalized for dissolved Ag (for dissolution, see Figure 3). * - significantly (p<0.05) different from EC_50_ of AgNO_3_.

**Figure 6:**
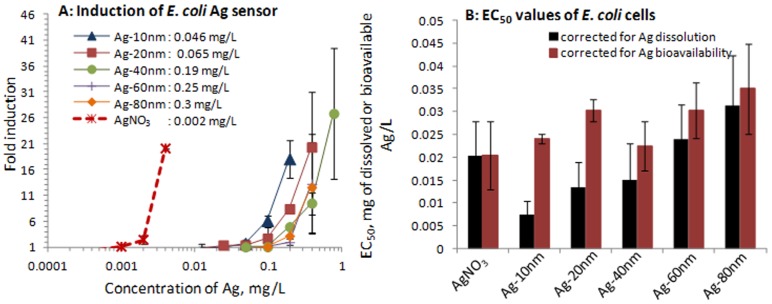
Response of *E. coli* sensor to subtoxic concentrations of Ag formulations and bioavailability-corrected EC_50_ values. (A) Induction of bioluminescence in Ag-inducible *E.coli* bioreporter strain by AgNO_3_ and 10–80 nm Ag NPs. Concentration of different Ag formulations at 2-fold induction is shown; (B) 4-h EC_50_ of AgNO_3_ and Ag NPs, corrected for dissolved Ag (see Figure 3) or bioavailable Ag, calculated from bioluminescence induction of Ag bioreporter strain (see panel A).

As expected, toxicity of Ag NPs increased with decreasing particle size. The difference in toxicity of 80 nm Ag NPs and 10 nm Ag NPs was the biggest for *D. magna* (about 20 fold) and the smallest for mammalian fibroblasts (1.7-fold) (Figure S6). To quantify the role of Ag ions in the toxicity of Ag NPs, we normalized the Ag NP EC_50_ values for all the test organisms/cells to dissolved Ag ions (Figure 5). The dissolution-corrected EC_50_ values of Ag 20, 40, 60 and 80 nm NPs were similar to those of AgNO_3_, indicating that in case of these particles, toxicity was induced by released Ag ions. However, the toxicity of 10 nm Ag NPs was higher than predicted by soluble Ag-ions and this effect was statistically significant (Figure 5). It seems that 10 nm Ag NPs have additional not-dissolution driven toxic properties compared to 20–80 nm Ag NPs. Interestingly, similar observation, dissolution independent toxicity of ≤10 nm Ag NPs was recently published by Gliga et al. [28]. Consequently, it appears that the real size threshold for novel biological effects of particles is closer to <20 nm and not 100 nm as suggested in the current ‘nano’ legislation [29]. In order to determine the factors inducing these additional size-dependent toxic effects, we hypothesized that due to their small size, the 10 nm Ag NPs may induce ROS and/or induce increased bioavailability of silver.

d **Evaluation of the size-dependent reactive oxygen species (ROS)-production potential of Ag NPs.** In order to study the factors behind the surplus toxic effects of 10 nm Ag NPs, we first tested whether enhanced production of extracellular ROS could contribute to their ‘excess’ toxicity. Moreover, induction of ROS is, in addition to ion release theory, one of the best acknowledged mechanisms of toxic action of Ag NPs [30]. Although AgNO_3_ at concentrations >1 mg/L, 10 nm Ag NPs at concentrations >10 mg/L and 20 nm Ag NPs at concentrations >50 mg/L increased the fluorescence of ROS indicator dye 2,7-H_2_DCFDA, these concentrations were much higher than the EC_50_ values of AgNO_3_ or the respective Ag NPs (Table 3, Figure 4). At the EC_50_ concentrations for most of our test organisms no ROS-induced increase in 2,7-H_2_DCFDA fluorescence was detected (Table S3). Thus, ROS were most probably not involved in Ag NPs toxicity and did not explain the excess toxicity of 10 nm Ag NPs. Slight induction of the ROS-indicator dye was registered at the EC_50_ concentrations of AgNO_3_ (1.7 mg Ag/L) and 10 nm Ag NPs (16.9 mg Ag/L) to mammalian fibroblasts (Table S3) indicating that ROS may be involved in silver toxicity for these cells.

e **Analysis of the effect of particle size in particle-cell direct contact for bioavailability of silver.** Since abiotic ROS did not explain the enhanced toxicity of 10 nm Ag NPs (see above) and our previous studies [31] have shown that the main driver for Ag NP toxicity is the contact between the organism/cell and NPs, we next analyzed direct cellular bioavailability of the Ag NPs. According to our previous studies, bioavailability of metals from NPs [31] and other solid matrices (soils, sediments; [32] [33] [34] [35]) is not always equal to metal dissolution. Specifically, in close contact with the particulate matter, cells may release and import higher concentrations of metals than are dissolved in abiotic conditions. Thus, we analyzed Ag bioavailability from Ag NPs and AgNO_3_ (an ionic control) using *E. coli* biosensor cells that exhibit induced bioluminescence in response to subtoxic concentrations of Ag ions that enter the cells [17]. Similarly to the tendency in Ag NPs' toxicity (Figure 4) and dissolution (Figure 3), the bioluminescent response of sensor bacteria increased with decreasing Ag NP's size. The latter indicates that more Ag ions entered bacterial cells in case of the same mass of smaller Ag NPs compared to larger ones (Figure 6A). When the 4h EC_50_ values of differently sized Ag NPs for *E. coli* (Figure 4: upper panel) were normalized to bioavailable Ag, measured by *E. coli* Ag-sensor bacteria, no 10 nm NP threshold effect was observed any more (Figure 6B). Thus, the toxic effects of all the Ag NPs, including 10 nm ones, could be completely explained by intracellular bioavailable Ag. Although our data showed that the toxic effects of Ag NPs to bacteria were due to intracellular bioavailable silver, we do not have analogous experimental proof to claim that this was also the case for other test organisms. In addition, even if the toxicity was due to bioavailable Ag ions, we do not have enough information on mechanisms by which these Ag ions became bioavailable. As shown in this study and also in our previous studies [31], the direct contact between metallic NPs and cells is essential for increased bioavailability of metals. However, it is still unclear whether the NPs enter the cells and then become bioavailable or dissolve extracellularly in close vicinity of cell surface before entering the cells and becoming bioavailable to the living cells. In the scientific literature both, uptake of NPs by microbial cells [36] [37] [24] as well as increased dissolution of NPs in the vicinity of microbial cells have been suggested [38] [39] [40]. In case of three marine phytoplankton species exposure to polystyrene NPs increased the production of exopolymers [41] which indicates that NPs may significantly interfere with cellular physiology. However as the interaction of NPs with living cells and *vice versa* is a dynamic process, determination of the exact sequence of cellular events triggered by NP exposure remains to be elucidated.

## Conclusions

In conclusion, the toxicity of Ag NPs was strictly dependent on NPs dissolution but the bulk dissolution explained the toxicity of 20–80 nm Ag NPs but not of 10 nm Ag NPs. This discrepancy was clarified by using *E. coli* Ag-biosensor that showed equal intracellular bioavailability of nanosilver, whatever the particle size. Therefore, 10 nm and smaller particles seem to interact with cells and become more bioavailable either by dissolving in the close vicinity of the outer cell surface or inside the cells. These results indicate that in order to obtain more efficient Ag NPs antimicrobials, one has to stream towards <10 nm particles. More research is needed to find out the exact mechanism driving the enhanced bioavailability and toxicity of ≤10 nm Ag particles. Whether Ag ion bioavailability is driving the toxicity of ≤10 nm Ag NPs also to other test organisms than bacteria remains also to be elucidated.

## Supporting Information

Figure S1 Number of papers registered in Thomson Reuters ISI Web of Science on search terms “toxic* AND nano*” and “size-dependent toxic* AND nano*” in different years. Search was performed on December 8, 2013.
(TIF)

Figure S2 **Number of published papers on toxicity of nanosilver to different organisms.** Search was performed in Thomson Reuters ISI Web of Science (all years). Search term “effect of size and silver and nano*” that yielded altogether 3363 papers was refined by additional search terms referring to organisms/organism groups which are indicated on x-axis. Most information was available for bacteria (270 papers in total) and most of the bacteria-related papers concerned *Escherichia coli* (246 papers) – a model bacterium widely used in hygienic and/or medical studies in design of novel antimicrobials. Search was performed on December 8, 2013.
(TIF)

Figure S3 **Variability of currently published toxicity data on nanosilver.** Toxicity of Ag NPs varies remarkably: analysis of the literature data (from [4]) showed that the data varied even within the same organism group: remarkable differences were 500-fold in the case of bacteria, 4240-fold in the case of algae and 275-fold in the case of mammalian cells *in vitro*. It was suggested that this high variability in nanosilver toxicity was due to differences in NPs as well as in testing conditions.
(TIF)

Figure S4 **Schematic representation of experiments conducted in this study.**
(TIF)

Figure S5 **Organismal differences in sensitivity to Ag NPs and AgNO_3_.** Average values for all the five studied sizes of Ag NPs 10, 20, 40, 60 and 80 nm are presented (see also Table 3). Nominal concentrations-based EC_50_ values are shown. Note the logarithmic Y-scale.
(TIF)

Figure S6 **Organismal differences in sensitivity to differently sized Ag NPs.** (A) Nominal EC_50_ values for different test organisms. Note the logarithmic Y-scale; (B) Ratio between EC_50_ of Ag-80 nm and EC_50_ of Ag-10 nm.
(TIF)

Table S1 **Toxicity of Ag NPs and Ag ions to bacteria, yeast, algae, crustaceans, fish and mammalian cells** ***in vitro*** **.** The values are selected and summarized from [4]. Altogether 119 L(E)C_50_ or MIC values were found for Ag NPs and 72 L(E)C_50_ or MIC values were found for Ag ions. Crustaceans (daphnids), algae and fish—the aquatic test organisms proposed for the classification and labelling of chemicals by EU REACH regulation—proved the most sensitive groups of organisms in respect to the toxic action of Ag NPs.
(DOCX)

Table S2 **Dissolution of Ag NPs (%) in test media used for different toxicity assays.**
(DOCX)

Table S3 **Abiotic production of reactive oxygen species (ROS) by Ag NPs of different sizes and AgNO_3_.** The potential for abiotic ROS was measured at EC_50_ concentrations (Table 3) of the different Ag formulations.
(DOCX)
