# Supplementary figures and images for: Size-Dependent Toxicity of Silver Nanoparticles to Bacteria, Yeast, Algae, Crustaceans and Mammalian Cells In Vitro
Source: PLoS One. 2014 Jul 21;9(7):e102108. doi: 10.1371/journal.pone.0102108 (PMC4105572; doi:10.1371/journal.pone.0102108)

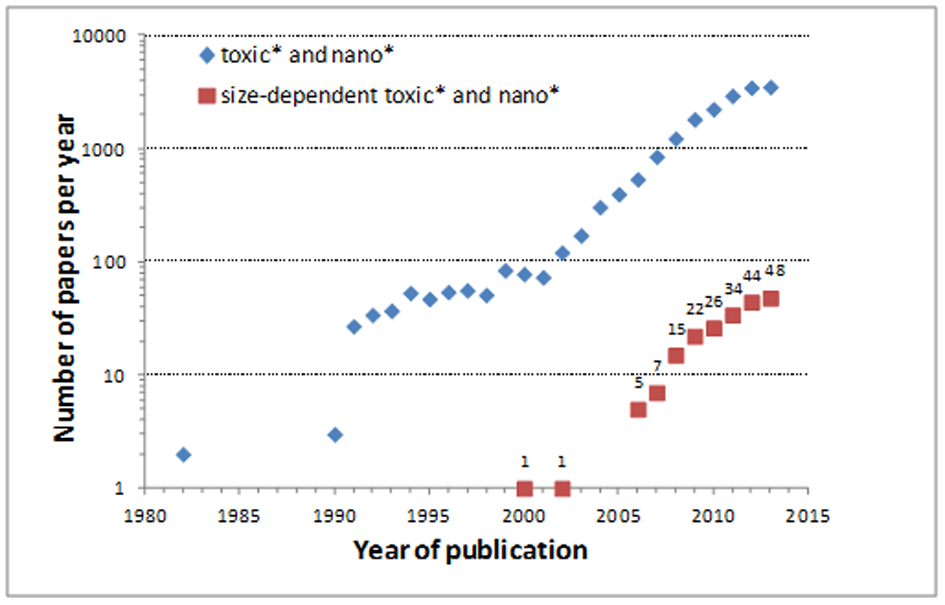

Supplement: Figure S1 — Number of papers registered in Thomson Reuters ISI Web of Science on search terms “toxic* AND nano*” and “size-dependent toxic* AND nano*” in different years. Search was performed on December 8, 2013. (TIF) [file pone.0102108.s001.tif]

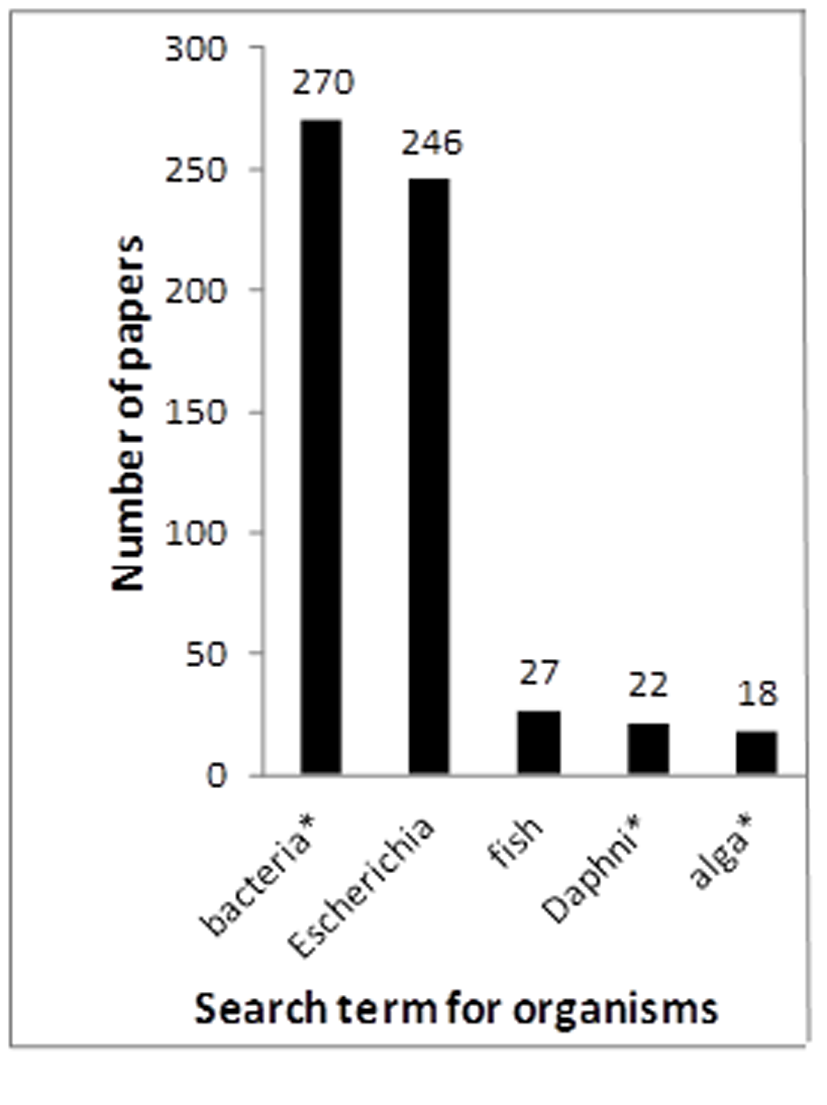

Supplement: Figure S2 — Number of published papers on toxicity of nanosilver to different organisms. Search was performed in Thomson Reuters ISI Web of Science (all years). Search term “effect of size and silver and nano*” that yielded altogether 3363 papers was refined by additional search terms referring to organisms/organism groups which are indicated on x-axis. Most information was available for bacteria (270 papers in total) and most of the bacteria-related papers concerned Escherichia coli (246 papers) – a model bacterium widely used in hygienic and/or medical studies in design of novel antimicrobials. Search was performed on December 8, 2013. (TIF) [file pone.0102108.s002.tif]

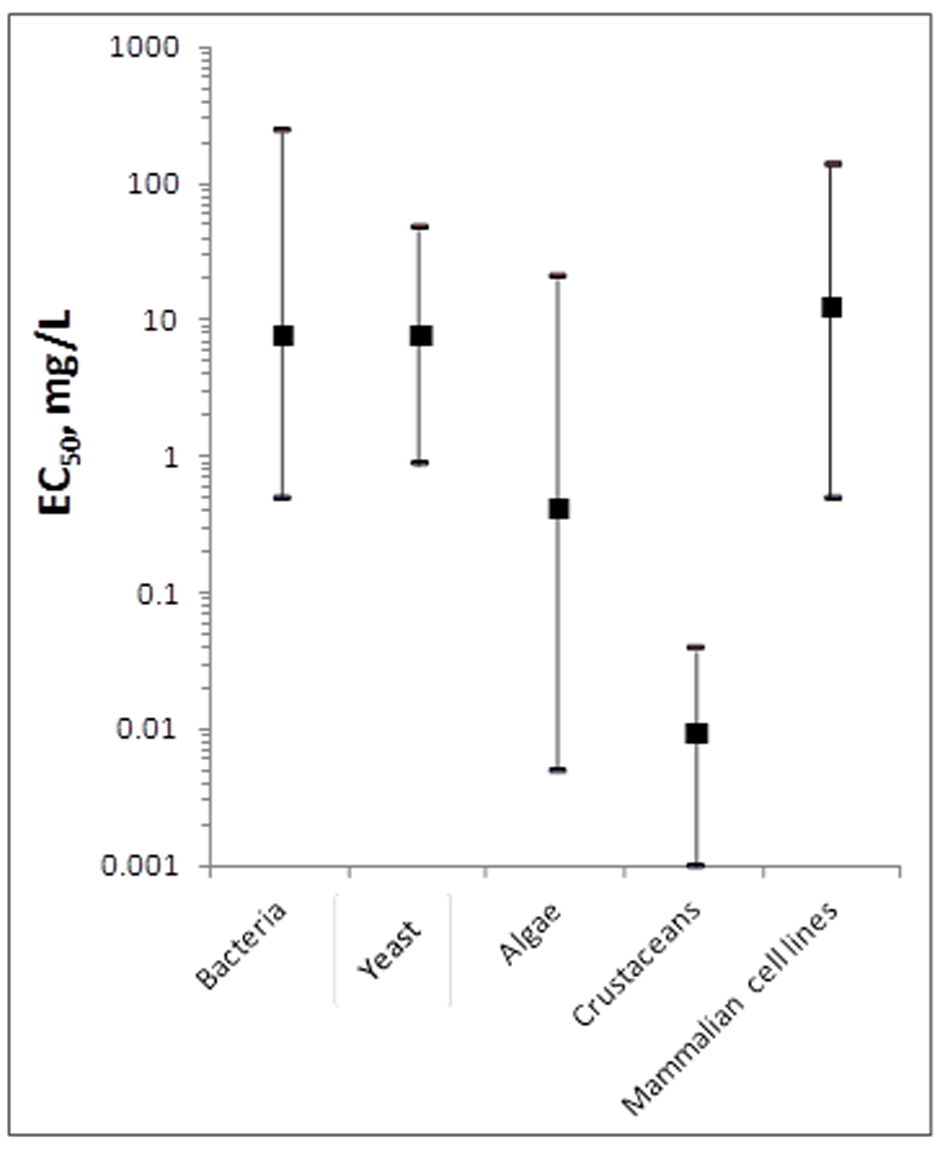

Supplement: Figure S3 — Variability of currently published toxicity data on nanosilver. Toxicity of Ag NPs varies remarkably: analysis of the literature data (from [4]) showed that the data varied even within the same organism group: remarkable differences were 500-fold in the case of bacteria, 4240-fold in the case of algae and 275-fold in the case of mammalian cells in vitro. It was suggested that this high variability in nanosilver toxicity was due to differences in NPs as well as in testing conditions. (TIF) [file pone.0102108.s003.tif]

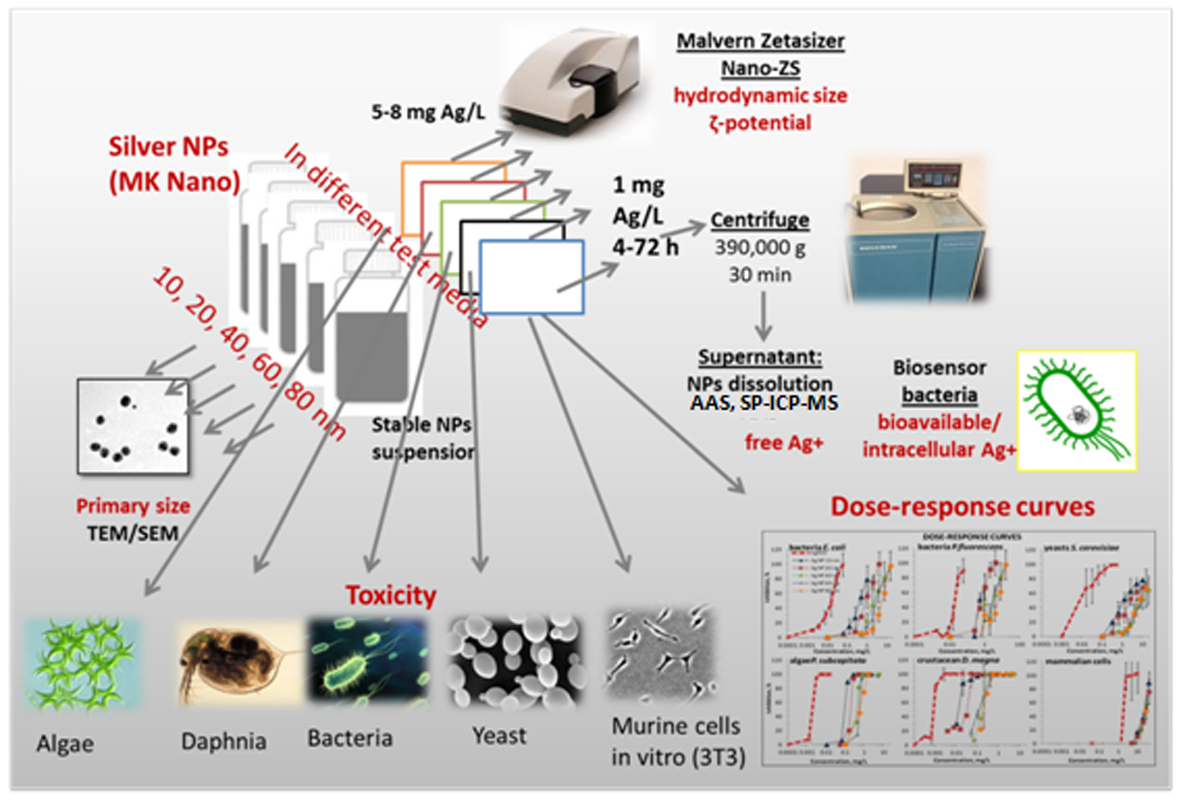

Supplement: Figure S4 — Schematic representation of experiments conducted in this study. (TIF) [file pone.0102108.s004.tif]

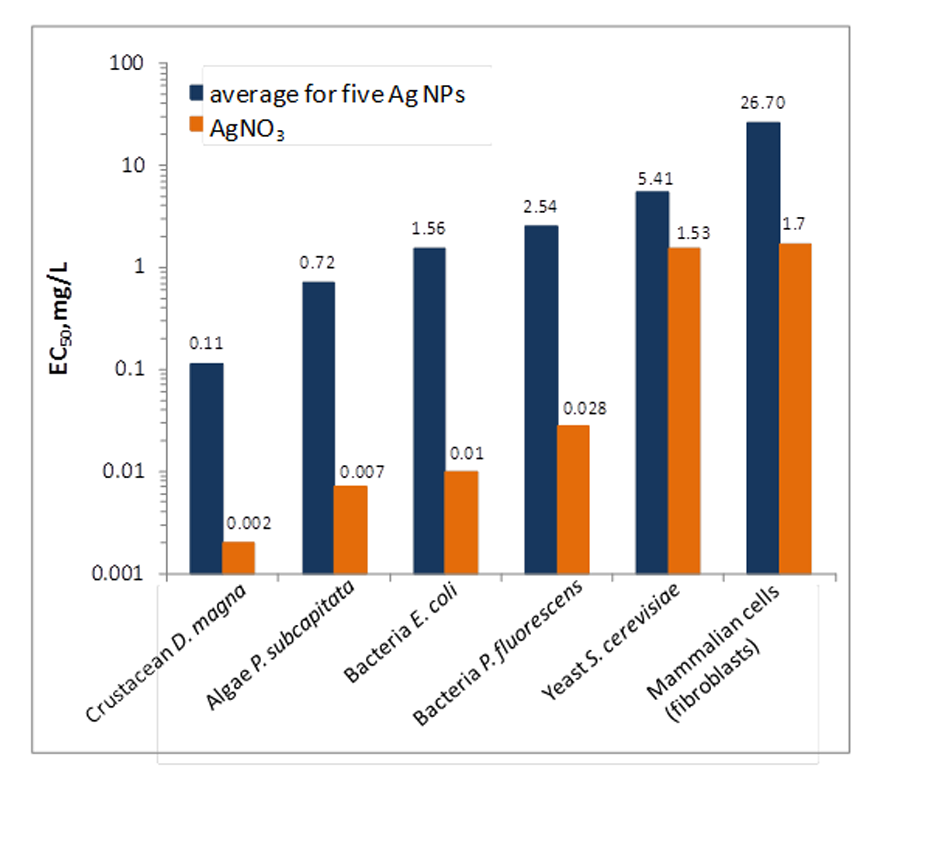

Supplement: Figure S5 — Organismal differences in sensitivity to Ag NPs and AgNO3. Average values for all the five studied sizes of Ag NPs 10, 20, 40, 60 and 80 nm are presented (see also Table 3). Nominal concentrations-based EC50 values are shown. Note the logarithmic Y-scale. (TIF) [file pone.0102108.s005.tif]

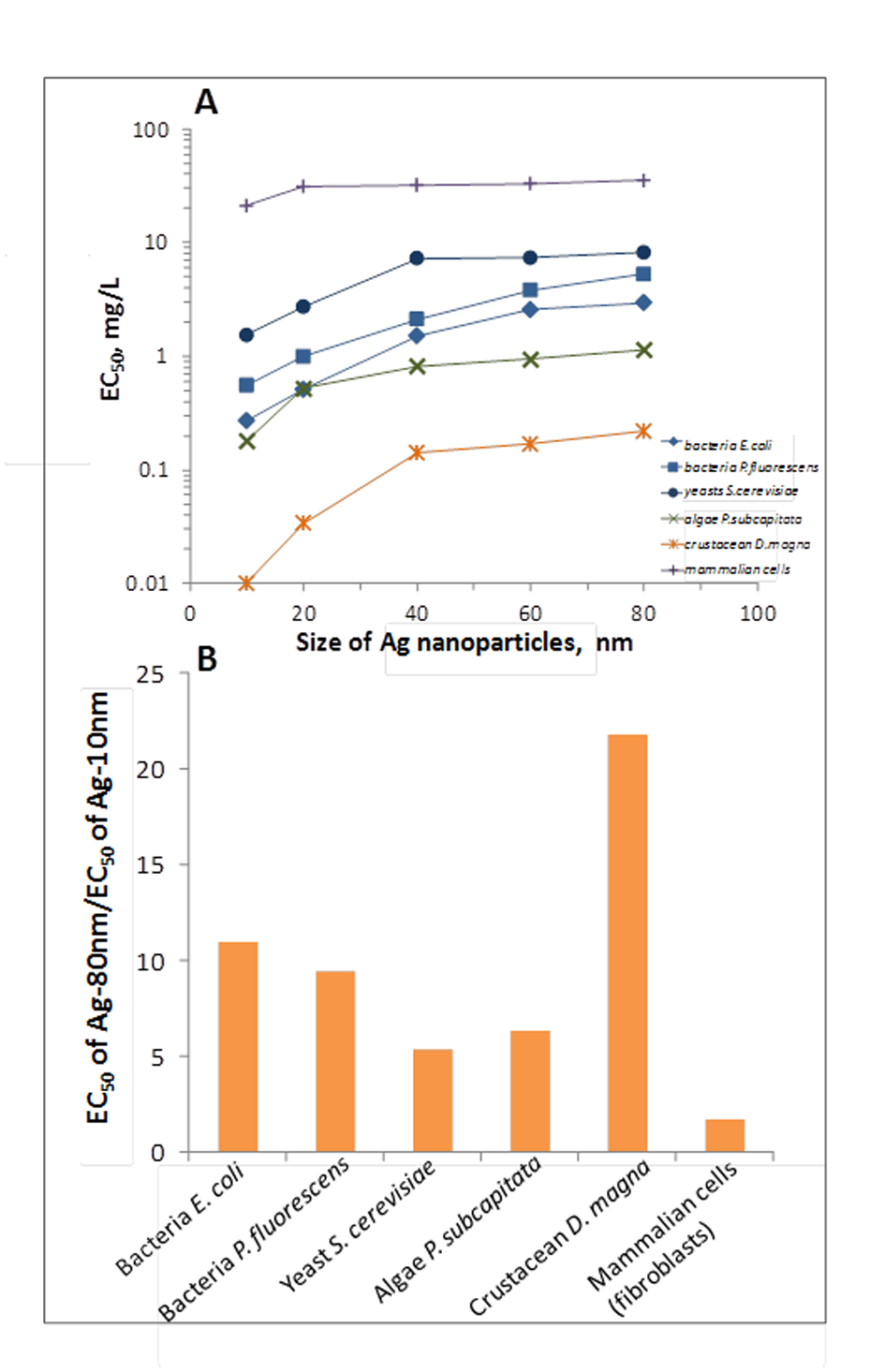

Supplement: Figure S6 — Organismal differences in sensitivity to differently sized Ag NPs. (A) Nominal EC50 values for different test organisms. Note the logarithmic Y-scale; (B) Ratio between EC50 of Ag-80 nm and EC50 of Ag-10 nm. (TIF) [file pone.0102108.s006.tif]
