# Supplementary material for: Size-Dependent Toxicity of Silver Nanoparticles to Bacteria, Yeast, Algae, Crustaceans and Mammalian Cells In Vitro
Source: PLoS One. 2014 Jul 21;9(7):e102108. doi: 10.1371/journal.pone.0102108 (PMC4105572; doi:10.1371/journal.pone.0102108)
Supplement: Table S1 — Toxicity of Ag NPs and Ag ions to bacteria, yeast, algae, crustaceans, fish and mammalian cells in vitro . The values are selected and summarized from [4]. Altogether 119 L(E)C50 or MIC values were found for Ag NPs and 72 L(E)C50 or MIC values were found for Ag ions. Crustaceans (daphnids), algae and fish—the aquatic test organisms proposed for the classification and labelling of chemicals by EU REACH regulation—proved the most sensitive groups of organisms in respect to the toxic action of Ag NPs. (DOCX) [file pone.0102108.s007.docx]

**Table S1. Toxicity of Ag NPs and Ag ions to bacteria, yeast, algae, crustaceans, fish and mammalian cells *in vitro.*** The values are selected and summarized from [[4](#_ENREF_1)]. Altogether 119 L(E)C_50_ or MIC values were found for Ag NPs and 72 L(E)C_50_ or MIC values were found for Ag ions. Crustaceans (daphnids), algae and fish—the aquatic test organisms proposed for the classification and labelling of chemicals by EU REACH regulation—proved the most sensitive groups of organisms in respect to the toxic action of Ag NPs.

|  | Median L(E)C_50_^a^ or MIC^b^ value, mg of Ag/L,  (number of toxicity values used to derive the median value) | |
| --- | --- | --- |
|  | **Ag NPs** | **Ag^+^ ions**^c^ |
| Bacteria | 7.1 (46) | 3.3 (27) |
| Yeasts | 7.9 (14) | 2.2 (5) |
| Algae | 0.36 (17) | 0.0076 (10) |
| Crustaceans | 0.01 (17) | 0.00085 (8) |
| Fish | 1.36 (17) | 0.058 (4) |
| Mammalian cells *in vitro* | 11 (25) | 2.0 (18) |
| *The lowest L(E)C_50_ value* | *0.01* | *0.00085* |
| *Most sensitive organism group* | *Crustaceans* | *Crustaceans* |
| Total number of toxicity values for all test systems | 136 | 72 |

^a^ Half-lethal or half-effective concentration.

^b^ Minimal inhibitory concentration (used in case of bacteria).

^c^ toxicity values for Ag ions were collected for comparison, to evaluate the solubilisation-driven toxic effects of Ag NPs
