# Supplementary material for: Size-Dependent Toxicity of Silver Nanoparticles to Bacteria, Yeast, Algae, Crustaceans and Mammalian Cells In Vitro
Source: PLoS One. 2014 Jul 21;9(7):e102108. doi: 10.1371/journal.pone.0102108 (PMC4105572; doi:10.1371/journal.pone.0102108)
Supplement: Table S2 — Dissolution of Ag NPs (%) in test media used for different toxicity assays. (DOCX) [file pone.0102108.s008.docx]

**Table S2. Dissolution of Ag NPs (%) in test media used for different toxicity assays.**

|  | **Dissolved Ag, % ^a^** | | | |
| --- | --- | --- | --- | --- |
|  | UP ^b^ water  (4h) | AFW ^c^  (48 h) | Algal medium  (72 h) | Cell culture medium^d^  (24 h) |
| AgNO_3_ | 98.7 | 21.2 | 81.9 | 100 |
| Ag-10 nm | 1.32 | 1.39 | 1.55 | 6.94 |
| Ag-20 nm | 1.26 | 0.92 | 0.86 | 8.00 |
| Ag-40 nm | 0.81 | 0.43 | 0.75 | 5.16 |
| Ag-60 nm | 0.68 | 0.31 | 0.65 | 5.33 |
| Ag-80 nm | 0.65 | 0.28 | 0.61 | 7.52 |

^a^ Dissolved Ag was measured from supernatant of ultracentrifuged suspensions of Ag NPs (1 mg/L) or AgNO_3_ (0.01 mg/L)

^b^ ultrapure water, used in bacterial and yeast tests

^c^ AFW – artificial freshwater was used as the test medium for *Daphnia magna*

^d^ DMEM with high glucose, 10 % Newborn Calf Serum, 100 U/mL penicillin and 100 μg/mL streptomycin was used as the cell culture test medium
