# Supplementary material for: Size-Dependent Toxicity of Silver Nanoparticles to Bacteria, Yeast, Algae, Crustaceans and Mammalian Cells In Vitro
Source: PLoS One. 2014 Jul 21;9(7):e102108. doi: 10.1371/journal.pone.0102108 (PMC4105572; doi:10.1371/journal.pone.0102108)
Supplement: Table S3 — Abiotic production of reactive oxygen species (ROS) by Ag NPs of different sizes and AgNO3. The potential for abiotic ROS was measured at EC50 concentrations (Table 3) of the different Ag formulations. (DOCX) [file pone.0102108.s009.docx]

**Table S3. Abiotic production of reactive oxygen species (ROS) by Ag NPs of different sizes and** **AgNO_3_.** The potential for abiotic ROS was measured at EC_50_ concentrations (Table 3) of the different Ag formulations.

|  | **Fluorescence of 2,7- dichlorodihydrofluorescein diacetate, % of control^a^** | | | | | |
| --- | --- | --- | --- | --- | --- | --- |
| EC_50_ concentration for: | **AgNO_3_** | **Ag-10nm** | **Ag-20nm** | **Ag-40nm** | **Ag-60nm** | **Ag-80nm** |
| bacteria *E.coli* | 91.3 | 102.4 | 86.2 | 90.0 | 93.1 | 90.8 |
| bacteria *P.fluorescens* | 93.0 | 117.7 | 97.9 | 96.2 | 98.1 | 98.4 |
| yeast *S.cerevisiae* | 93.2 | 125.3 | 139.9 | 113.3 | 112.2 | 108.1 |
| crustacean *D.magna* | 90.0 | 74.2 | 74.6 | 75.7 | 83.5 | 81.7 |
| algae *P.subcapitata* | 90.7 | 85.6 | 86.5 | 82.7 | 86.6 | 84.8 |
| mammalian fibroblasts | 213.6 | 253.1 | 128.3 | 126.4 | 153.1 | 130.8 |

^a^ % fluorescence of 2,7- dichlorodihydrofluorescein diacetate in suspension of Ag formulation that corresponds to EC_50_ value of a specific organism compared to fluorescence in control (UP water). For comparison, H_2_O_2_ solution of 15 g/L resulted in 300% fluorescence of 2,7- dichlorodihydrofluorescein diacetate dye.
